# A commentary on ‘Perioperative versus postoperative calcium and vitamin D supplementation to prevent symptomatic hypocalcemia after total thyroidectomy: a randomized placebo controlled trial’

**DOI:** 10.1097/JS9.0000000000000351

**Published:** 2023-06-05

**Authors:** Zhe-Ming Zhao, Chun-Dong Zhang

**Affiliations:** Department of Gastrointestinal Surgery, The Fourth Affiliated Hospital, China Medical University, Shenyang, People’s Republic of China

*Dear Editor*,

With great interest, we read a recent prospective, double-blind, randomized, placebo-controlled trial reported by Sittitrai and his colleagues^1^. Among the 134 patients undergoing total thyroidectomy, 68 patients were orally administrated with calcium and vitamin D supplementation perioperatively (perioperative group), and 66 patients were orally administrated with calcium and vitamin D supplementation postoperatively (postoperative group). In this study, the researchers observed a significantly lower symptomatic hypocalcemia rate in the perioperative group than in the postoperative group (8.8 vs. 22.7%, *P*=0.033). Besides, they found a significantly lower mean calcium level in the perioperative group than in the postoperative group at 24 h (8.71 vs. 8.47 mg/dl, *P*=0.046) and 48 h (8.79 vs. 8.56 mg/dl, *P*=0.039) after surgery. Furthermore, they reported that the intravenous calcium requirement rate was lower in the perioperative group (2.9%) than the postoperative group (12.1%), although no significant difference between the two groups was observed (*P*=0.053). The researchers therefore clarified the significant advantages of perioperative oral calcium and vitamin D supplementation over postoperative oral calcium and vitamin D supplementation. We would like to share some opinions.

Postoperative hypocalcemia is one of the most common complications after total thyroidectomy. The occurrence of postoperative hypocalcemia was significantly correlated with the decrease of parathyroid hormone (PTH) after surgery^2,3^; therefore, the baseline preoperative PTH level may be crucial. In this study, the preoperative mean PTH level of the perioperative group (40.21 pg/ml) was 2.52 pg/ml higher than that of the postoperative group (37.69 pg/ml), although no significant difference was observed (*P*=0.217)^1^. The higher baseline preoperative PTH levels possibly reduced the risk of postoperative hypocalcemia, especially among those patients with higher PTH levels above the mean level of 40.21 pg/ml in the perioperative group.

Besides, operation-related indicators, that is operative duration and intravenous fluids (IVF), may correlate with the risk of postoperative hypocalcemia. Karunakaran *et al*.^4^ reported that operative duration >123 min and IVF >1085 ml significantly increased the risk of postoperative hypocalcemia. Furthermore, Falch *et al*.^5^ reported that operative duration >189 min increased by 80% risk of initial hypocalcemia [odds ratio (OR), 1.8, 95% CI, 1.2–2.6], and initial symptomatic hypocalcemia was highly associated with persistent hypocalcemia (OR, 40.9, 95% CI, 18.5–90.4). However, the data of such operation-related indicators are not reported in this study.

Furthermore, there is no detailed information about the use of steroids in this study. It has been reported that the preoperative use of steroids is effective in reducing postoperative hypocalcemia in patients undergoing thyroidectomy^6^. Notably, even a single dose of steroid, namely dexamethasone, reduced by 19% (95% CI, 11.1–27.7%) of symptomatic hypocalcemia rate than placebo^7^. The main findings of this study, therefore, await validation.

Overall, this study provides support for the idea that compared with postoperative oral calcium and vitamin D supplementation, perioperative oral calcium and vitamin D supplementation are more effective in preventing symptomatic hypocalcemia after total thyroidectomy. However, further research is still needed to verify the main findings of this study. Finally, we really appreciate the great efforts of Sittitrai and his colleagues in carrying out this very important study.

## Ethical approval

Not available.

## Sources of funding

This study received no funding.

## Author contribution

Z.-M.Z. and C.-D.Z.: manuscript preparation; C.-D.Z.: critical revision and submission.

## Conflicts of interest disclosure

The authors declare no conflicts of interest.

## Research registration unique identifying number (UIN)

1. Name of the registry: not available.
2. Unique Identifying number or registration ID: not available.
3. Hyperlink to your specific registration (must be publicly accessible and will be.
4. checked): not available.

## Guarantor

Chun-Dong Zhang.
